# Cancer odor in the blood of ovarian cancer patients: a retrospective study of detection by dogs during treatment, 3 and 6 months afterward

**DOI:** 10.1186/1471-2407-13-396

**Published:** 2013-08-26

**Authors:** György Horvath, Håkan Andersson, Szilárd Nemes

**Affiliations:** 1 Institute of Clinical Sciences, Department of Oncology, The Sahlgrenska Academy at University of Gothenburg, Gothenburg SE-41345, Sweden; 2 Department of Oncology, Sahlgrenska University Hospital, Gothenburg SE-41345, Sweden; 3 Regional Cancer Centre (West), Western Sweden Health Care Region, Sahlgrenska University Hospital, Gothenburg SE-41345, Sweden

**Keywords:** Trained dogs, Cancer odor in the blood, Ovarian carcinoma, Survival

## Abstract

**Background:**

In recent decades it has been noted that trained dogs can detect specific odor molecules emitted by cancer cells. We have shown that the same odor can also be detected in the patient’s blood with high sensitivity and specificity by trained dogs. In the present study, we examined how the ability of dogs to detect this smell was affected by treatment to reduce tumor burden, including surgery and five courses of chemotherapy.

**Methods:**

In Series I, one drop of plasma from each of 42 ovarian cancer patients (taken between the fifth and sixth courses of chemotherapy) and 210 samples from healthy controls were examined by two trained dogs. All 42 patients in Series I had clinical complete responses, all except two had normal CA-125 values and all were declared healthy after primary treatment. In Series II, the dogs examined blood taken from a new subset of 10 patients at 3 and 6 months after the last (sixth) course of chemotherapy.

**Results:**

In Series I, the dogs showed high sensitivity (97%) and specificity (99%), for detecting viable cancer cells or molecular cancer markers in the patients’ plasma. Indeed, 29 of 42 patients died within 5 years. In Series II, the dogs indicated positive samples from three of the 10 patients at both the 3- and 6-month follow-up. All three patients had recurrences, and two died 3–4 years after the end of treatment. This was one of the most important findings of this study. Seven patients were still alive in January 2013.

**Conclusions:**

Although our study was based on a limited number of selected patients, it clearly suggests that canine detection gave us a very good assessment of the prognosis of the study patients. Being able to detect a marker based on the specific cancer odor in the blood would enhance primary diagnosis and enable earlier relapse diagnosis, consequently increasing survival.

## Background

During the past two decades, an increasing number of authors have described cancer detection by dogs trained on various biological materials such as urine, breath, and stool [1-3]. Pickel [4] was the first to use tumor tissue from melanoma in the training of such dogs, and to our knowledge we are the only researchers to have used tissue from ovarian carcinomas or blood of patients with ovarian cancer [5,6]. We have previously shown that the odor emitted by cancer cells is also present in patients’ blood, and that trained dogs can detect it with high sensitivity and specificity [6]. We have also shown that dogs trained to recognize the odor of ovarian cancer could not recognize odors from other gynecological malignancies [5,6]. In addition, the dogs could not distinguish among different histopathological subgroups, stages or grades of ovarian carcinomas (including borderline tumors). The fact that the dogs could not recognize cancers other than ovarian cancer strongly suggests that different cancers have different characteristic smells, thus enabling both diagnosis and differential diagnosis. Moreover, the characteristic odor of ovarian carcinoma is likely organ-specific [5].

In addition to trained dogs, researchers have also used electronic noses to detect cancer-related volatile organic compounds in the headspace above malignant tissues [7,8]. These detection methods, however, had relatively low sensitivity and were not shown to be tumor-specific as the only comparisons made were versus healthy material. In their current form, electronic devices probably lack the sensitivity to distinguish a specific cancer from other cancers, which is a crucial requirement for practical use.

This specific odor of carcinomas is thus an important characteristic that is likely to play an important role in future early cancer diagnosis and also in disease monitoring. Our previous work [5,6] was based on tissue samples taken at primary surgery or blood taken before or immediately after surgery; the aim of those studies was primarily to investigate the possibility of using odor for screening and diagnosis of ovarian cancer. However, it may be useful to see how primary treatment (via the influence on tumor status) changes the production of cancer odor molecules. These changes may be mediated by various factors such as tumor burden, changes in malignant cell metabolism, tumor necrosis. In the future, the answer to this question may be crucial for odor-based monitoring in the follow-up of cancer.

The aim of the present study was to investigate how primary surgery and chemotherapy treatment affected the diagnosis of cancer odor in the blood of patients with different life expectancies based on their initial diagnosis. The study was conducted using two specially trained dogs that were used in our previous studies [6].

## Methods

### Ethics

This study was conducted in accordance with regulations of the Helsinki Declaration (1964) and conforms to the Regional Ethics Committee, Gothenburg.

### Dogs

Two dogs were used, Hanna, a 10-year-old black Giant Schnauzer (chip no. 967000000389928), and Lotti, a 6-year-old black Giant Schnauzer (chip no. 098100311386). The owner and handler is GH. The dogs live as family pets with the owner and his family. There is free access to fresh water all day, and feeding three times a day. The dogs spend several hours each day in a garden or on walks. Health checks are made at Värmdö Animal Clinic, Gustavsberg.

### Training

The training method has been described in detail elsewhere [5,6]. For 2 years prior to the present study, only once-a-week maintenance training was used. Each of the training sessions included 4–10 boxes [5], 0–3 of which contained cancer tissue or blood from patients with ovarian carcinoma. The setup was randomly selected before each session. This program allowed the dogs to be confronted with a different problem to solve in each training occasion. The dogs were rewarded only for a correct identification. Blood samples with >500 U/ml CA-125 values as an indicator of ovarian carcinoma [9] were used for training. One drop of the training sample was placed in a small plastic dish inside each box. The blood samples used during the training period were not used in the tests. The dogs were used in the experiment with the permission of the Regional Ethical Review Board in Gothenburg, license number: S-220-08. The dogs had free access to fresh water during training and testing hours. After 2 hours of work, the dogs were walked or had 20 minutes of free time.

### Patient selection

Patients were selected for inclusion in this retrospective study from the clinic and bio-bank databases. The latter contains blood and cancer tissue from patients with ovarian cancer. Material was collected after obtaining permission from the individual patient and was regulated by the treatment program for ovarian cancer in West Sweden. Patient selection first was made from the clinic database, then the results were correlated with the bio-bank database to obtain blood samples.

The major selection criterion applied to all study patients was clinical complete remission (CR) before the sixth (final) scheduled chemotherapy course. Patients selected for Series I were divided into 3 groups. Group A, included patients with 3 years of relapse-free survival, Group B included patients who had relapsed within 6 months after the last treatment session; and for Group C included patients who had relapsed between 1 and 2 years after treatment. A total of 66 patients with CR in the years 2001–2007 were selected in Series 1. All were from the Gothenburg area and were also in the bio-bank database. Forty-two patients had samples in a biobank corresponding to one of the three secondary selection criteria for inclusion in Group A, B or C. Selection for Series II was made using the biobank database only.

### Blood samples

Blood samples were collected from patients living in the Gothenburg area of West Sweden. The population is about 600,000. The treatment program for ovarian cancer in requires CA-125 analysis on two occasions. First, prior to or directly after primary surgery, and the second before the sixth course of chemotherapy treatment. However, as usual in the treatment program for ovarian carcinoma, CA-125 level was not included in the follow-up, although doctors have the option to check it. Blood samples with >500 U/ml CA-125 values were used for dog training, with one drop being placed in a small plastic dish inside each box. Blood samples used during the training period were not used in the tests.

### Reference blood samples

Material for the reference group was selected from the bio-bank database. Clinicopathological variables were not considered because our previous study results showed that they did not affect the dogs’ sensitivity of detection [5,6]. A total of 62 samples (42 for Series I and 20 for Series II), from different individuals, with CA-125 values >200 U/ml were randomly selected as reference material in Series I and II.

### Test blood samples in Series I

Forty-two samples were collected and used as test material in Series I. These blood samples were taken before the sixth course of chemotherapy. Patients were divided into three groups. Group A consisted of 13 patients who had a CR, 3-year relapse-free survival, and normal (<35 U/ml) CA-125values before their last treatment course. Group B consisted of 12 patients who had a CR, relapsed within 6 months, and all except 2 (61 U/ml; <200 U/ml) had CA-125 <35UI. Group C consisted of 17 patients with a CR, recurrence between 1 and 2 years, and normal CA-125 values. Tumor histopathology, stage and grade varied within the groups.

Blood samples with >500 U/ml CA-125 values were used for training, with one drop being placed in a small plastic dish inside each box. Blood samples used during the training period were not used in the tests.

### Test blood samples in Series II

Samples were taken 3 and 6 months after the sixth, final chemotherapy course. Unfortunately, we could not follow patients in Series I, Group A because none of them had blood in the blood bank. We collected blood from 10 other patients who were followed regularly. Median donor age was 65 years (range, 38–78 years).

### Control blood samples

Control samples were collected during the 2 years between 2007 and 2009, mostly from female medical staff volunteers in Gothenburg. Inclusion criteria were that the patients felt healthy, were not pregnant and were free of gynecological disease. Control and test materials were not age-matched. Younger persons were consciously chosen as the source of control samples to reduce the risk of the presence of asymptomatic of ovarian cancer. Thus, both the control and test groups had samples from pre-and postmenopausal women.

### Sample preparation

Blood samples were collected in EDTA tubes, and then centrifuged at 3000 rpm for 10 min with plasma pots over the small plastic tubes. After centrifugation, the plasma was divided into two parts, one for CA-125 analysis and the other for subsequent experiments. The latter part was kept at −80°C in the tumor bank (Ethical Committee license number: S-220-08, Regional Ethical Review Board in Gothenburg). Control plasma samples were processed and stored identically to the targets. However, tubes with control blood were stored separately. Median donor age was 45 years (range, 29–65 years).

### Test design

Tests were carried out in a double-blind fashion as previously described [6]. To summarize, both test leader and handler were blinded to the location of the target samples, and were present in the test location only when the dogs were working. The dogs were tested in two series. Series I covered 4 days (2 days per occasion), while Series II covered 2 days. Ten runs were performed on each day, except for one day in Series I when 11 runs were carried out. Each run included seven boxes, placed in a circle about 2 m apart from each other. Each box [5,6] contained a drop of plasma; five contained control materials, one contained a test sample, and one contained a reference sample. Reference materials were taken before, or shortly after the primary operation, and thus had a high concentration of odor molecules. The placement of the target and reference boxes was changed by an assistant between each run. Each box was cleaned with alcohol between runs. The tests were documented on paper and DVD by the test leader and one assistant [6].

### Dog responses

A positive response was defined as indicating the target box by scratching with the foreleg, lying down and sniffing it (and not indicating the control samples). A negative response was defined as sniffing and indicating a control box and not indicating the target. An uncertain response was defined as stopping at the box, smelling it, scratching at it, and possibly barking, but going straight on and not lying down.

### Treatment of ovarian carcinomas

In line with the standard treatment program in West Sweden, patients were treated by total hysterectomy, bilateral salpingo-oophorectomy, omentectomy, multiple peritoneal biopsies, and peritoneal washings with cytology. Approximately 4 weeks after primary surgery the first carboplatin, cyclophosphamide, and epirubicin combination was given. A total of six courses were administered at 4-week intervals.

### Statistical methods

The raw data were summarized as sensitivity (the conditional probability of the dog indicating cancer when the condition was present) and test specificity (the conditional probability of the dog ignoring a sample from a healthy donor). Sensitivity and specificity give insight into the general classification ability of the dogs.

The positive and negative predictive probability that the test would give the correct diagnosis were also calculated. Point estimates were calculated with 95% confidence intervals [10]. Both sensitivity and specificity were expressed as proportions, thus standard techniques for proportions could be applied for statistical inference. Confidence intervals were based on the normal approximation, $p\pm 1.96\sqrt{p\left(1-p\right)/n}$ where p is the estimated sensitivity, respective specificity and n is the number of test runs.

The test runs are best described as having a hypergeometric distribution. The probability of a perfect test run (i.e., finding the test sample and ignoring the controls) by chance was 1/6, and the probability of performing all the runs without making any errors follows the binomial distribution. Reference samples were not included in the statistical analyses.

## Results and discussion

### Series I

Between them, the dogs correctly indicated all 42 reference samples, giving a sensitivity of 100%. Lotti correctly indicated 41 of the 42 test samples and wrongly indicated 2 of the 210 controls, giving a sensitivity of 97% and a specificity of 99%. Hanna correctly indicated 41 of the 42 test samples and made no erroneous identifications among the controls, giving a sensitivity of 97% and a specificity of 100% (Table 1). The combined results for both dogs showed a sensitivity of 97% and a specificity of 99% (Table 2).

**Table 1:** Dogs responses

			**Series I**			**Series II (3 m)**			**Series II (6 m)**	
		**Ref.**	**Cancer**	**Control**	**Ref.**	**Cancer**	**Control**	**Ref.**	**Cancer**	**Control**
Lotti	Yes	42	41	2	10	6	2	10	6	5
	No	0	1	208	0	4	48	0	4	45
Hanna	Yes	42	41	0	10	8	3	10	10	3
	No	0	1	210	0	2	47	0	0	47

Ref.: reference samples.

**Table 2:** Sensitivity and specificity measures in Series I (both dogs together)

	**Estimate**	** 95% CI**	
		**Lower**	**Upper**
Sensitivity	0.976	0.908	0.995
Specificity	0.995	0.980	0.999
For any particular test result, the probability that it will be:			
Positive	0.166	0.135	0.202
Negative	0.833	0.797	0.864
For any particular positive test result, the probability that it is:			
True positive	0.976	0.908	0.995
False positive	0.023	0.004	0.091
For any particular negative test result, the probability that it is:			
True negative	0.995	0.980	0.999
False negative	0.004	0.000	0.0190

Each dog missed one test sample—one from Group B and one from Group C; both patients died of cancer—but still had a generally high sensitivity and specificity. There should be no doubt in assuming that, in the majority of patients, the number of characteristic odor molecules would have been limited compared with the reference material, and that it was this that led to the failures to identify test samples. In contrast, the dogs identified all reference samples correctly.

It is likely that the completion of surgery and chemotherapy reduced the number of cancer cells in the patients’ bodies, thereby reducing the number of odor molecules in their blood. Moreover, it seems likely that there were large individual quantitative differences in characteristic odor molecules in different samples. There will have been a wide range in the number of molecules in the samples, but the dogs were unable to signal quantitative differences; all they could do was to signal a positive or a negative result. However, the results are interesting because there were a number of patients who had radical surgery and subsequently received five courses of chemotherapy. All patients had clinical CR before the sixth course, as evaluated by palpation under general anesthesia and in some cases completed with a CT scan, and all except two had normal CA-125 values.

Generally, doctors do not know how many patients will have residual cancer cells after complete clinical remission is declared, and it is unknown whether the final treatment after this will kill any remaining cells. In fact, our results suggest that almost all of the patients in our study had viable cancer cells, and the majority (n=29) of the 42 patients died of their disease. In Group A, two patients died of intercurrent disease, one of ovarian carcinoma between 4 and 5 years after the treatment was finished, and one died of probable cancer, although without a diagnosed relapse. Nine patients survived until the 5-year follow-up. One of the dead patients in Group A had stage I/A, two had stage I/C and the fourth had Stage III. Of the nine surviving patients, two had Stage II/A, four had Stage I/A, and three had Stage I/B.

In Group B, all patients died within 2 years. In this group one patient had stage I/A, one had I/C and the remaining patients had stage III–IV. In Group C, one patient survived for 4 years relapse-free, but was then lost to follow-up. The remaining 16 patients died between 16 months and 5 years after the end of treatment. Two patients had stage I/C, one had IIB and 12 patients had Stage III or IV. A patient who was lost to follow-up had stage III disease.

We do not have information on clinicopathological features such as stage, tumor grade, histology, age and menopausal status of the individual patients included in Series I. However, our previous studies [5,6] clearly showed that when dogs were trained to recognize the smell of ovarian cancer, those variables did not affect sensitivity. Furthermore, although the study shows detection of cancer odor to be a very good prognostic factor, depending on the size of the group and the material selected, comparison with other known prognostic factors cannot be done.

On the other hand, it is also possible that surviving patients had residual living cancer cells between their fifth and sixth courses of treatment, but far fewer cells than patients who died of their disease, and the odor molecules from those cells were detected by the dogs.

It is difficult to discuss our results on a broader basis, because to our knowledge there are no other published studies that have used blood samples with trained dogs and related the findings to survival. However, our results strongly suggest a great need for a more sensitive marker than is currently available to ensure the safety of patients and increase the overall survival of ovarian cancer patients.

### Series II

All 20 reference samples were correctly indicated by both dogs, giving a sensitivity of 100%.

### Three-month test samples

Lotti correctly indicated six of 10 test samples (one of which was uncertain) and wrongly indicated two of 50 controls, giving a sensitivity of 60% and a specificity of 96%. Hanna correctly indicated eight of 10 test samples (one of which was uncertain) and wrongly indicated three of 50 controls, giving a sensitivity of 80% and a specificity of 94% (Table 1). The combined results for both dogs showed a sensitivity of 70% and a specificity of 95% (Table 3).

**Table 3:** Sensitivity and specificity measures in Series II, 3 months (both dogs together)

	**Estimate**	** 95% CI**	
		**Lower**	**Upper**
Sensitivity	0.7	0.456	0.871
Specificity	0.95	0.881	0.981
For any particular test result, the probability that it will be:			
	0.158	0.100	0.238
Negative	0.841	0.7612	0.899
For any particular positive test result, the probability that it is:			
True positive	0.736	0.485	0.898
False positive	0.2631	0.101	0.514
For any particular negative test result, the probability that it is:			
True negative	0.940	0.870	0.975
False negative	0.059	0.024	0.129

### Six-month test samples

Lotti correctly indicated six of 10 test samples (one of which was uncertain) and wrongly indicated five of 50 controls, giving a sensitivity of 60% and a specificity of 90%. Hanna correctly indicated all 10 test samples (although four of the 10 selections were uncertain), and wrongly indicated three of 50 controls, giving a sensitivity of 100% and a specificity of 94% (Table 1). The combined results for both dogs showed a sensitivity of 80% and a specificity of 92% (Table 4).

**Table 4:** Sensitivity and specificity measures in Series II, 6 months (both dogs together)

	**Estimate**	** 95% CI**	
		**Lower**	**Upper**
Sensitivity	0.8	0.557314	0.933894
Specificity	0.92	0.843855	0.962321
For any particular test result, the probability that it will be:			
Positive	0.2	0.134742	0.284927
Negative	0.8	0.715073	0.865258
For any particular positive test result, the probability that it is:			
True positive	0.666667	0.446926	0.835734
False positive	0.333333	0.164266	0.553074
For any particular negative test result, the probability that it is:			
True negative	0.958333	0.890714	0.986569
False negative	0.041667	0.013431	0.109286

During the 2-day test for Series II, both dogs appeared to be under stress and generally unsettled; they barked, made various unusual sounds, and often turned back and tried to go in the opposite direction between the boxes. We suspected that this could have been due to an unusually low concentration of odor molecules in the test samples. We tested the dogs in the interval between the 2 test days without a test sample in the arrangement, and they performed the search as usual. The following day, when the test samples were included again, the anxious and insecure behavior returned. However, the fact that identification of reference samples was 100% correct and the specificity was high (as it was in our previous studies) suggests that the overall results are correct.

We selected samples from patients who were relapse-free during the first 3 years after treatment. This choice of time period was based on the fact that most recurrences are diagnosed in the first 3 years [11]. Patients were selected from our database and since the disease may relapse even after the first 3 years after treatment, we also checked the patient case records in January 2013 (Table 5). The 3- and 6-month test samples of two patients (No. 8 and 9), were both clearly indicated by both dogs. For one additional patient (No. 3), the 3-month sample was indicated by both dogs while the 6-month sample was clearly indicated by Hanna and uncertainly indicated by Lotti. All three patients had recurrences, and two of them died 3–4 years after the end of treatment. The January 2013 record check showed that two of the 10 patients (No. 8 and 9) included in Series II who were thought to be recurrence-free during the first 3 years after treatment, had in fact relapsed a few months before 3 years had passed. The reason they were categorized as having no relapse was that the database had not been updated at the time of patient selection, and their current recurrence was not known to us.

**Table 5:** Tumor characteristics, dog responses, and survival for patients in Series II

**No**	**Test samples 3 m/6 m**	**Diagnosis date**	**Stage**	**Histopathology**	**Grade**	3 months* Lotti Hanna	6 months* Lotti Hanna	**Case record Jan 2013**
1	4842/5095	Feb 2009	III/C	Seropapillary	2	O O	X #	Alive/relapse-free
2	3932/4799	June 2008	III/C	Seropapillary	3	O X	X X	Alive/relapse-free
3	406/1018	Nov 2005	III/C	Adenocarcinoma	3	X X	# X	Relapse 2008/Died 2009
4	9807/108	Jan 2005	II/C	Adenocarcinoma	2	O X	O #	Alive/relapse-free
5	8647/8955	Dec 2003	III/C	Clear cell	2	X X	O X	alive/relapse-free
6	10704/1328	Dec 2005	II/C	Seropapillary	3	# O	O #	Alive/relapse-free
7	451/1065	Sept 2005	III/C	Undifferentiated	4	X X	O #	Relapse 2009/alive
8	3835/6750	Feb 2000	III/C	Seropapillary	3	X X	X X	Relapse 2002/died 2005
9	6132/7872	Jan 2004	II/C	Seropapillary	2	X X	X X	Relapse 2006/alive
10	4926/5214	Aug 2008	II/B	Seropapillary	2	O #	X X	Alive/relapse-free

* = all CA-125 values were <35UI.

Dog responses:

X = positive indication as cancer; O = no indication; # = uncertain indication.

Six-month test samples from two patients (No. 2 and 10) were clearly indicated by the two dogs, but the patients remained relapse-free 4 years after completion of treatment. The dogs’ indications in those cases were fairly consistent, which may imply an increased risk of recurrence in future years.

The remaining indications were consistent with patient survival, although in some cases (e.g., No. 1 and 4) there were suggestions by uncertain indication behavior that there may be a few viable cancer cells remaining in the body. It is likely that in several cases the concentration of typical odor molecules was near the lower limit of canine detection ability. To estimate what this limit might be, we have previously published results showing that one dog (Hanna) was repeatedly able to identify with certainty a piece of fatty abdominal wall containing about 20 microscopically-verified ovarian cancer cells [6]. It is impressive how this very low limit of detection allows dogs to signal probable future recurrences that would not be diagnosed by other methods for another 2–3 years. This is the most important result of the present study.

The dogs were able to indicate small numbers of living cancer cells with high sensitivity and specificity in a large group of ovarian cancer patients. To our knowledge; this is the first study to highlight the importance of characteristic odor molecules in the blood of ovarian cancer patients as a prognostic marker. Previously, McCulloch et al. described one patient with breast cancer in remission who was identified by dogs as having cancer [2]. Detection of odor in the blood, currently only possible with trained dogs, can allow for early and long-term prediction of survival. An early diagnosis of primary or recurrent disease may also significantly improve the patient’s survival.

## Conclusion

In summary, although our results are based on a limited number of patients, they clearly show that canine detection gave us a very good opportunity to assess the prognosis of the study patients Being able to detect a marker based on the specific cancer odor in the blood would enhance both primary diagnosis and relapse diagnosis. An instrument with a sensitivity and specificity close to that of the trained dogs used here is necessary for future oncology.

## Competing interests

This work was partly supported by Royal Canin AB, Sweden. The authors had no other relevant affiliations or financial involvement with any organization or entity having a financial interest in or financial conflict with the subject matter or materials discussed in the manuscript, apart from those disclosed.

No writing assistance was used in the production of this manuscript.

The figures and tables presented here are original and have not been presented earlier.

## Authors’ contributions

GH: Project leader, study planning, working with dogs, conducting tests, manuscript writing; HA: Collection of data, manuscript writing; Sz N: Statistical analysis, manuscript writing. All authors read and approved the final manuscript.

## Pre-publication history

The pre-publication history for this paper can be accessed here:

http://www.biomedcentral.com/1471-2407/13/396/prepub
